# Antimicrobial activity and toxicological evaluation of didecyldimethyl-ammonium chloride complexed with fatty alcohol ethoxylate

**DOI:** 10.14202/vetworld.2026.1724-1746

**Published:** 2026-04-29

**Authors:** Olga Gruznova, Dmitry Gruznov, Natalya Pavlova, Gulizar Shcherbakova, Ekaterina Shuteeva, Nikolay Kuvshinchikov, Nikolay Popov, Valeriya Tyumentseva, Viktoriya Pchelkina, Anton Lobanov, Polina Tyubaeva, Ivetta Varyan

**Affiliations:** 1 Laboratory of Veterinary Sanitation, All-Russian Research Institute of Veterinary Sanitation, Hygiene and Ecology – Branch of Federal State Budget Scientific Institution “Federal Scientific Center – K.I. Skryabin, Ya.R. Kovalenko All-Russian Research Institute of Experimental Veterinary Medicine, Russian Academy of Sciences”, Moscow, Russia; 2 Laboratory of Liquid-Phase Oxidation, N.N. Semenov Federal Research Center for Chemical Physics, Russian Academy of Sciences, Moscow, Russia; 3 Laboratory of Veterinary Medicine and Environmental Safety in Beekeeping, All-Russian Research Institute of Veterinary Sanitation, Hygiene and Ecology – Branch of Federal State Budget Scientific Institution “Federal Scientific Center – K.I. Skryabin, Ya.R. Kovalenko All-Russian Research Institute of Experimental Veterinary Medicine, Russian Academy of Sciences”, Moscow, Russia; 4 Laboratory of Pharmacology and Toxicology, All-Russian Research Institute of Veterinary Sanitation, Hygiene and Ecology – Branch of Federal State Budget Scientific Institution “Federal Scientific Center – K.I. Skryabin, Ya.R. Kovalenko All-Russian Research Institute of Experimental Veterinary Medicine, Russian Academy of Sciences”, Moscow, Russia; 5 Laboratory of Sanitary Microbiology, All-Russian Research Institute of Veterinary Sanitation, Hygiene and Ecology – Branch of Federal State Budget Scientific Institution “Federal Scientific Center – K.I. Skryabin, Ya.R. Kovalenko All-Russian Research Institute of Experimental Veterinary Medicine, Russian Academy of Sciences”, Moscow, Russia; 6 Experimental Clinic-Laboratory of Biologically Active Substances of Animal Origin, V.M. Gorbatov Federal Research Center for Food Systems, Russian Academy of Sciences, Moscow, Russia; 7 Department of General Chemistry, Moscow Pedagogical State University, Moscow, Russia; 8 Department of Physical Chemistry of Synthetic and Natural Polymer Compositions, Emanuel Institute of Biochemical Physics, Russian Academy of Sciences, Moscow, Russia; 9 Academic Department of Innovational Materials and Technologies Chemistry, Plekhanov Russian University of Economics, Moscow, Russia.

**Keywords:** acute toxicity, antimicrobial activity, disinfectants, didecyldimethylammonium chloride, fatty alcohol ethoxylate, membrane disruption, surfactants, veterinary sanitation

## Abstract

**Background and Aim:**

Effective disinfection is critical for controlling pathogenic and opportunistic microorganisms in livestock environments. Didecyldimethylammonium chloride (DDAC), a quaternary ammonium compound, is widely used due to its strong antimicrobial properties; however, improving its efficacy without increasing toxicity remains a key challenge. This study aimed to evaluate the antimicrobial activity of DDAC in combination with fatty alcohol ethoxylate (FAEO8) and to assess the toxicological safety of the resulting complex preparation.

**Materials and Methods:**

The antimicrobial activity of DDAC, FAEO8, and DDAC–FAEO8 was evaluated against *Staphylococcus aureus*, *Escherichia coli*, and *Salmonella* Typhimurium using minimum inhibitory concentration (MIC) determination, inhibition zone diameter assays, scanning electron microscopy, potassium ion (K^+^) leakage analysis, and membrane potential assays. Toxicological evaluation included acute and sub-acute oral toxicity studies in rats and dermal irritation/corrosion tests in rabbits, conducted in accordance with international and national guidelines.

**Results:**

The DDAC–FAEO8 complex exhibited significantly enhanced antimicrobial activity compared to DDAC alone, with MIC reductions of 44.4%, 30.8%, and 26.7% against *S. aureus*, *E. coli*, and *S*. Typhimurium, respectively. At concentrations ≥2× MIC, the complex inhibited >99% of microbial growth and demonstrated prolonged bacteriostatic effects. Microscopic analysis revealed pronounced structural damage and cell lysis, while K^+^ leakage and membrane depolarization assays confirmed disruption of bacterial membrane integrity. FAEO8 alone showed no bactericidal activity but did contribute to changes in membrane permeability. Toxicological assessment indicated that the complex preparation was non-lethal at doses up to 2000 mg/kg, with no significant pathological alterations observed. Subacute exposure showed minimal physiological changes, and dermal testing confirmed that the diluted formulation (40 mg/mL) was non-corrosive, with only mild, reversible irritation.

**Conclusion:**

The combination of DDAC with FAEO8 enhances antimicrobial efficacy through membrane disruption mechanisms while maintaining low toxicity. This additive interaction highlights the potential of DDAC–FAEO8 as an effective and safe disinfectant for veterinary applications.

## INTRODUCTION

The rapid development of animal husbandry has been driven by increasing consumer demand for animal-derived products [1–4]. Nevertheless, infectious diseases remain a major obstacle to the advancement of this agricultural sector. One of the most important approaches for combating these diseases is the timely and effective disinfection of livestock facilities. Therefore, the quality of disinfection measures largely depends on the proper selection of disinfectants [5–9].

Among effective disinfecting agents, quaternary ammonium compounds (QACs) have been widely used for their pronounced antimicrobial activity against both Gram-positive and Gram-negative microorganisms [10–13]. QACs are water-soluble and do not deform treated surfaces. These properties provide certain advantages over preparations from other chemical classes [14]. However, QACs are also regarded as a serious environmental concern because their residues can accumulate in wastewater and soil. In addition, they may promote the emergence of resistant microorganisms, thereby disturbing microbial ecology [15–18]. Resistance of microorganisms to this class of compounds is thought to arise through several mechanisms, including porin modification, regulatory overexpression of efflux pumps, enzymatic degradation [19], and the acquisition of resistance genes [20]. The latter mechanism was clearly demonstrated by Bayrakal *et al* [21], in which QAC-resistant genes of *Staphylococcus aureus* (*qacA/B, qacC, qacG, qacH, qacJ*, and *smr*) were identified in meat and dairy production facilities. In contrast, Yang *et al*. [22] did not detect induced resistance of *Escherichia coli* strains isolated from activated sludge to 16 different QACs. Despite these concerns, the production volume of QACs remains high. This was especially notable during the COVID-19 pandemic, when the virucidal activity of QACs was confirmed [15, 23, 24].

In addition, several studies have focused on the synthesis of new QACs. For example, Kuruca and Akarsu [14] developed silane-QACs based on (3-chloropropyl)triethoxysilane using QACs with carbon chain lengths of C12, C14, C16, and C18. Gunther *et al*. [25] reported the synthesis of QACs with multiple cationic groups, known as multiQACs. Although the exact mechanism of action of QACs has not been fully elucidated, it is generally assumed that the positively charged nitrogen atom electrostatically interacts with negatively charged residues of teichoic and lipoteichoic acids, thereby destabilizing the membrane. Subsequently, hydrophobic substituents become inserted into the membrane matrix, leading to cytoplasmic leakage and cell lysis. Moreover, some QACs may also target DNA and pyridoxal-dependent enzymes [26–28].

Didecyldimethylammonium chloride (DDAC), characterized by two long alkyl chains, is a representative member of the QAC class [29, 30]. DDAC has shown strong antimicrobial activity against Gram-positive and Gram-negative microorganisms [31–34]. It has been registered as a disinfectant by the United States Environmental Protection Agency [35]. Together with other QACs, DDAC is widely used in healthcare and food industries, as well as for the disinfection of veterinary facilities [11, 32, 36–38]. For instance, DDAC is a component of several commercial disinfectants, including DimonCare (AdvaCare Pharma, USA), 50% and 80% DDAC (EasyChem, China), 80% DDAC (Chemical Private Limited, India), 50% and 80% DDAC (Rugao Wanli Chemical Industry Co., Ltd., China), and 50% DDAC (Ecotech, Russia).

Because decontamination includes not only disinfection but also cleaning, the presence of cleaning properties in a disinfectant can improve its overall effectiveness [7]. Surface-active agents have been shown to perform well for this purpose [39–41]. Although some surfactants exhibit antimicrobial activity, others lack strong biocidal properties [42–44]. Certain surfactants, such as Tween 80, Tris, Triton X-100, and Brij 96, are known to enhance the inhibitory activity of antibiotics by affecting bacterial membrane integrity [45–47]. Conversely, suppression of bactericidal bioavailability by surfactants has also been reported [48].

The influence of surfactants on the antimicrobial activity of disinfectants when used in combination remains a poorly studied yet highly relevant issue. The present study focused on comparing the antimicrobial activity of free DDAC with that of DDAC in the presence of fatty alcohol ethoxylate with eight moles of ethylene oxide (FAEO8), which is readily soluble in water and possesses good cleaning and emulsifying properties [49, 50]. To our knowledge, there are currently no reports explaining the mechanism by which the bioavailability of disinfectants may increase or decrease in the presence of this surfactant. The closest available study examined the intrinsic bacteriostatic effect of a homologous series of alcohol ethoxylates with the same head-group size (E6) but differing in the number of carbon atoms in their tail group, ranging from 10 to 16 [51].

Despite the extensive use of QACs such as DDAC in veterinary sanitation, several important limitations remain insufficiently addressed. Although DDAC demonstrates strong antimicrobial activity, enhancement of its efficacy often requires higher concentrations, which may contribute to environmental accumulation and potential toxicity concerns. In addition, the emergence of microbial tolerance and resistance to QACs continues to raise concerns regarding their long-term effectiveness in livestock production systems. While surfactants are commonly incorporated into disinfectant formulations to improve cleaning efficiency, their influence on the antimicrobial performance of QACs has not been fully elucidated. In particular, the role of FAEO8 in modulating membrane permeability, influencing disinfectant bioavailability, and altering bacterial susceptibility remains poorly understood. Furthermore, previous studies have largely evaluated antimicrobial activity and toxicological properties separately, with limited integration of both efficacy and safety within a single experimental framework. Importantly, there is a lack of systematic evidence on the interaction between DDAC and FAEO8, particularly regarding their combined antimicrobial mechanism, membrane-targeting effects, and toxicological profile under *in vivo* conditions against representative microorganisms such as *S. aureus*, *E. coli*, and *S*. Typhimurium. Therefore, a comprehensive investigation that addresses both the enhanced antimicrobial efficacy and the safety of such combined formulations is warranted.

The present study aimed to comprehensively evaluate the antimicrobial efficacy and toxicological safety of a combined formulation of DDAC and FAEO8. Specifically, the study compared the antimicrobial activity of DDAC alone and in combination with FAEO8 against representative Gram-positive and Gram-negative bacteria, including *S. aureus*, *E. coli*, and *S*. Typhimurium. In addition, the study aimed to elucidate the mechanism underlying the enhanced antimicrobial effect of the combined preparation by investigating membrane disruption through scanning electron microscopy (SEM), K^+^ leakage analysis, and membrane potential assays. The nature of the interaction between DDAC and FAEO8 was further assessed using fractional inhibitory concentration index analysis. Furthermore, a comprehensive toxicological evaluation, including acute and subacute toxicity studies and dermal irritation/corrosion testing, was conducted to ensure the formulation’s safety for practical veterinary use. By integrating antimicrobial and toxicological assessments, this study aimed to determine whether the DDAC–FAEO8 combination can provide an effective and low-toxicity disinfectant suitable for veterinary surveillance facilities.

## MATERIALS AND METHODS

### Ethical approval

Ethical approval for this study was obtained from the Ethical Committee of the All-Russian Research Institute of Veterinary Sanitation, Hygiene and Ecology, Moscow, Russia (protocol no. 02/25-RRIVSHE, approved on May 17, 2025). All animal procedures were conducted in strict accordance with Federal Law No. 498-FL “On Responsible Treatment of Animals” (Russian Federation, 2018), Directive 2010/63/EU of the European Parliament and of the Council on the protection of animals used for scientific purposes, and the Guide for the Care and Use of Laboratory Animals (National Academy Press, USA, 2011).

The toxicological component of the study involved Wistar male rats and New Zealand White female rabbits. Before inclusion, all animals underwent veterinary inspection and quarantine, and only clinically healthy animals were enrolled. Housing, feeding, environmental control, and daily welfare monitoring were maintained under standard laboratory conditions throughout the experimental period. Animal grouping was performed using random sampling based on predefined age and body weight criteria, and observers were blinded to group allocation in all toxicological assessments. Humane endpoints were applied throughout the study, and rats in the acute oral toxicity experiment were euthanized humanely using CO_2_ asphyxiation at the end of the observation period before necropsy.

The acute oral toxicity study, sub-acute toxicity study, and acute dermal irritation/corrosion test were performed according to internationally recognized guidelines, including OECD Test Guideline No. 423, OECD Test Guideline No. 407, and OECD Test Guideline No. 404, respectively, as well as the relevant Russian regulatory standards for disinfectant safety assessment. All efforts were made to minimize animal suffering, reduce the number of animals used, and ensure that procedures were scientifically justified and ethically proportionate to the study’s objectives.

For the microbiological and mechanistic components of the work, no human participants were involved. Bacterial strains were handled in a Biosafety Level 2 laboratory in accordance with institutional biosafety requirements.

### Study period and location

The study was conducted from March 17, 2025, to September 20, 2025, at the All-Russian Research Institute of Veterinary Sanitation, Hygiene and Ecology (accredited by the Federal Service for Veterinary and Phytosanitary Surveillance of Russia) in accordance with the institute’s schedule and funding receipt. SEM was performed at the V. M. Gorbatov Federal Research Center for Food Systems.

### Materials

**Figure 1:**
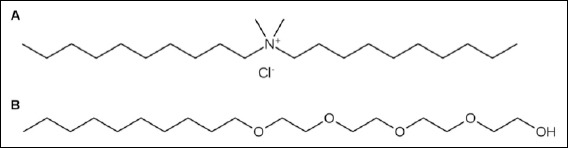
The DDAC (A) and FAEO8 (B) structures. The structures were created using the ChemDraw software.

DDAC 50% (v/v), Ecotech, Moscow, Russia, CAS 7173-51-5, C22H48ClN, purity ≥95%, Mw 362.08 g/mol, stable at temperatures from 5°C to 30°C); fatty alcohol ethoxylate (FAEO8, Lutensol® AO8, BASF, Ludwigshafen am Rhein, Germany, Mw 334.5 g/mol); valinomycin (Merck, Darmstadt, Germany, CAS 2001-95-8, C54H90N6O18, purity ≥98%, Mw 1111.32 g/mol); 3,3′-dipropylthiadicarbocyanine iodide (DiSC3(5), Merck, Darmstadt, Germany, CAS 53213-94-8, C25H27IN2S2, purity ≥98%, Mw 546.54 g/mol); sterile physiological solution (0.9% NaCl, Khimikom, Nizhny Novgorod, Russia); meat-peptone agar (MPA, Khimikom, Nizhny Novgorod, Russia); salt meat-peptone agar (sMPA, Khimikom, Nizhny Novgorod, Russia); and meat-peptone broth (MPB, Khimikom, Nizhny Novgorod, Russia) were used in this work. The structures of DDAC and FAEO8 are shown in Figure 1.

### Stock solutions preparation

The stock solutions of DDAC and FAEO8 were prepared in sterile double-distilled water (ddH_2_O) to obtain concentrations of 40.0 mg/mL and 7.0 mg/mL, respectively. A complex solution (DDAC-FAEO8) containing the indicated substances in similar concentrations was also prepared. Dissolution was carried out using a magnetic stirrer (Dlab, model MS-H280-Pro, Beijing, China) for 15 min (500 rpm, 22°C ± 1°C). All stock solutions were filtered through a polytetrafluoroethylene (PTFE) membrane filter with a pore diameter of 0.22 μm (Millipore, Darmstadt, Germany) and stored at 5°C in the dark, ensuring stability for 1 year. The concentration of DDAC in solutions was confirmed spectrophotometrically using a ultraviolet–visible (UV–Vis) spectrophotometer (PE5400UF, Ekroskhim, St. Petersburg, Russia).

### Bacterial strains and cultivation conditions

In this research, we employed the 209-P strain of *S. aureus*, the 1257 strain of *E. coli*, and the ATCC 14028 strain of *S*. Typhimurium. The characteristics of the strains are shown in Table 1.

**Table 1:** Microorganism strains’ characteristics.

Bacterial strains	Isolation source	Antibiotic susceptibility	Antibiotic resistance
*Staphylococcus aureus* 209-P	clinical isolate	methicillin, oxacillin, streptomycin, cefazolin, ceftriaxone, cefepime, amoxicillin/clavulanate, vancomycin, linezolid, gentamicin, fosfomycin, clindamycin, tetracycline, oxytetracycline	penicillin
*Escherichia coli* 1257	clinical isolate	imipenem, meropenem, fosfomycin, amikacin, gentamicin, nitrofurantoin, furazidine, levofloxacin, and oxytetracycline	amoxicillin, sulfamethoxazole-trimethoprim, amoxicillin-clavulanate, ciprofloxacin
*Salmonella* Typhimurium ATCC 14028	heart and liver tissues from 4-week-old chicken	fosfomycin, gentamycin, imipenem, kanamycin, mezlocillin, moxifloxacin, neomycin, nitrofurantoin, norfloxacin, ofloxacin, penicillin G, pipemidic acid, piperacillin/tazobactam, polymyxin, quinupristin/dalfopristin, tetracycline, ticarcillin	bacitracin, clindamycin, lincomycin, linezolid, nystatin, oxacillin, teicoplanin, vancomycin

These strains were obtained from the cell culture repository maintained by the All-Russian Research Institute of Veterinary Sanitation, Hygiene and Ecology. Their identity was confirmed by matrix-assisted laser desorption/ionization time-of-flight mass spectrometry (MALDI-TOF MS) using a Bruker Daltonik MALDI Biotyper (Bruker Daltonik GmbH, Bremen, Germany).

All experiments with the indicated strains were carried out in a Biosafety Level 2 laboratory.

For the daily cultivation of *S. aureus*, we utilized a slanting MPA and incubated it at 37°C for 24 h in a dry-air incubator (TV-80-1 model, State Ryazan Instrument Plant, Ryazan, Russia). The incubation period for *E. coli* and *S*. Typhimurium was 12 h.

To determine the optical density (OD), the bacteria were recultured in sterile MPB (Khimikom, Nizhny Novgorod, Russia). Suspensions containing 10^9^ colony-forming units per milliliter (CFU/mL) were prepared from daily cultures in a physiological saline solution. The suspensions were standardized according to the industry turbidity standard for determining total microorganism concentrations (BAK-10 kit, Ormet, Yekaterinburg, Russia) and then subjected to serial dilutions with 10-fold increments from 10^8^ to 10^5^ CFU/mL using sterile saline solution. Suspensions were used in experiments within 60 min of preparation and standardization (intraday variability did not exceed 5%). The resulting concentrations were verified using a UV–Vis spectrophotometer (model PE5400UF, manufactured by Ekroskhim) at a wavelength of 600 nm.

### Determination of the minimum inhibitory concentrations (MICs) and calculation of the fractional inhibitory concentration index

To determine the MICs for DDAC, FAEO8, and their combination (DDAC-FAEO8), test solutions were prepared by serial dilution. Initially, the stock solutions were diluted 2-fold to concentrations ranging from 125 to 0.06125 μg/mL. After establishing the MIC ranges, dilutions from 1.0 to 0.1 μg/mL (in 0.1 μg/mL increments) were used for *S. aureus*. Concentrations of 0.05 μg/mL and 0.025 μg/mL were included to determine the 50% effective concentration (EC50). Preparations dilutions for *E. coli* ranged from 2.6 to 0.4 μg/mL (dilution increments of 0.2 μg/mL), and from 3.0 to 0.8 μg/mL (dilution increments of 0.2 μg/mL) for *S*. Typhimurium. Subsequently, 180 μL of a bacterial suspension at 10^8^ CFU/mL was added to each well of a sterile 96-well microplate (Deltalab, Barcelona, Spain), along with 20 μL of the respective test solution. The samples were incubated for 24 h at 37°C with stirring on an orbital shaker (Joanlab, model SK-20, Zhejiang, China) at 80 rpm. OD600 was measured using a microplate reader (Mindray, model MR-96A, China) before and after incubation. Suspensions of bacterial cells without any pretreatment, but with the addition of solvent (ddH_2_O), served as a negative control group. The MICs were defined as the concentration at which no change in OD was observed relative to the pre-incubation measurement. The experiments were carried out in three technical replicates (standard deviation [SD] < 5%) [52, 53].

To determine the nature of the interaction between DDAC and FAEO8, the fractional inhibitory concentration index (FICI) was calculated using the following formula:

FICI = (MICDDAC-FAEO8 / MICDDAC) + (MICDDAC-FAEO8 / MICFAEO8)

where MICDDAC-FAEO8 is the MIC of complex preparation DDAC-FAEO8; MICDDAC is the MIC of DDAC; MICFAEO8 is the MIC of FAEO8.

FICI < 0.5 is considered to indicate synergy; 0.5 < FICI < 1.0 is considered additive; 1.0 < FICI < 4.0 is considered indifferent; and an FICI > 4.0 is considered antagonism [54, 55].

### Microorganisms’ survival evaluation

To determine the viability of *S. aureus*, *E. coli*, and *S*. Typhimurium in the presence of DDAC, FAEO8, and DDAC-FAEO8, the optical density (OD600 nm) of pure sterile MPB, MPB contaminated with the microorganism but without the addition of the preparation, MPB with the addition of the preparation but without contamination, and contaminated MPB containing the preparation were measured. The bacterial survival (%) after 24 h incubation was calculated using the following formula:

Survival (%) = [(ODtest solution – ODcorresponding control) / (ODassay growth control – ODsterility control)] × 100

where ODtest solution is optical density of contaminated MPB with the addition of the preparation; ODcorresponding control is MPB containing the preparation; ODassay growth control is optical density of MPB contaminated with a microorganism without the addition of the preparation; ODsterility control is optical density of intact MPB [56].

### Inhibition zone diameters determination (IZDs)

The antibacterial activity of DDAC, FAEO8, and DDAC-FAEO8 was determined using an MPA by measuring the IZD formed around wells containing the test solution. First, 1 mL of the microbial suspension at a concentration of 10^5^ CFU/mL was added to Petri dishes containing sterile nutrient medium and spread evenly over the surface using a sterile disposable spatula. Wells were then made in the center of an MPA dish using a sterile punch, and 100 μL of the preparation solutions (2× MIC) was added to each well. Next, the samples were placed in a thermostat (37°C) and incubated for 168 h with daily recording of the diameter of the growth inhibition zone (mm). Temperature and humidity levels in the thermostat were controlled using an electronic thermohygro logger (iLoggers iLog.Mth Light, Moscow, Russia). The diameters were measured with a digital caliper (ADA Mechanic model 150 A00379, Shenzhen, China). When measuring irregular IZD, the diameter was averaged along two mutually perpendicular axes. The seeding density was assessed by counting the colonies grown using a Scan 3000 AI automatic counter (Interscience, Cantal, France). The sterility of the nutrient medium was confirmed by the absence of microbial growth on dishes that were neither contaminated nor treated with preparations. The experiments were carried out in three technical replicates (SD < 5%) [53, 57].

### SEM

0.5 mL of *S. aureus*, *E. coli*, and *S*. Typhimurium cultures (10^9^ CFU/mL) were added to a test tube containing 5 mL of sterile MPB. The test tube was placed in a thermostat set to 37°C to increase bacterial mass. 100 µL of the test solutions (2× MIC dose) was added to the test tubes. The control sample was not exposed to preparations. Next, incubation was carried out for 12 h in a dry-air incubator at 37°C. 1.75 mL of each sample was placed in test tubes and centrifuged at 14,100 × *g* for 15 min in a centrifuge (Eppendorf MiniSpin plus, Eppendorf, Hamburg, Germany). The supernatant liquid was removed. 1.75 mL of sterile ddH_2_O was added to the sediment. The samples with dense sediment were placed in an ultrasonic bath (Sapfir, AZ Engineering, Moscow, Russia) for 20 min at 25°C. Then the centrifugation cycle was repeated to remove the supernatant. 0.5 mL of sterile ddH_2_O was added to the resulting centrifugate, and the mixture was vortexed (Vortex V-1 plus, BioSan, Riga, Latvia) until a homogeneous suspension was obtained. 50 µL of the suspension was evenly distributed in a thin layer over the surface of the cover glass. The coverslips were placed in a sealed desiccator (DWK Life Sciences, Mainz, Germany). Fixation was carried out with 25% (v/v) glutaraldehyde (BASF AG, Ludwigshafen am Rhein, Germany) vapor in a tightly closed desiccator at room temperature (22°C ± 2°C) for 15 min in a fume hood (Korsa, Moscow, Russia). Then the samples were dehydrated in ethanol (JSC Rosspirtprom, Moscow, Russia) at increasing concentrations: 30%, 50%, 70%, and 96% (v/v), with each solution kept for 10 min. After dehydration, the samples were dried in a fume hood until the solvent had completely evaporated. The cell morphology was studied using a Coxem EM-30 Plus microscope (Coxem, Daejeon, Korea). For this purpose, the fixed sample was secured to a sample holder using carbon double-sided adhesive tape and coated with a thin layer of gold using an SPT-20 ion coating system (Coxem, Daejeon, Korea) at 100 s and 5 mA. The samples were scanned at ×4000 magnification [58–60].

Lysed (deformed) cells were counted in 20 microscopic fields for each sample. The following formula was used to calculate the percentage of lysed cells:

Lysed cells (%) = (number of lysed cells / total number of cells) × 100

The experiments were carried out in three biological replicates (three independent bacterial cultures).

### Analysis of K+ leakage

*S. aureus*, *E. coli*, and *S*. Typhimurium were cultured on slanted MPA for 24-48 h at 37°C to obtain a daily culture containing 10^9^ CFU/mL. The bacterial suspension was then centrifuged at 15000 rpm for 30 min, after which the supernatant was removed, and the sediment was washed twice with 25 mL of 0.1 M MgCl_2_ solution. 10 mL of McIlvain buffer (0.1 M citric acid and 0.2 M sodium hydrogen phosphate, pH 7.0) containing the studied preparations at a concentration of 2× MIC was added to the sediment. The control samples (negative control) were not exposed to the preparations. Valinomycin was used as a positive control and was contained in the McIlvain buffer at a concentration of 0.1 μM.

The absence of pH changes during the analysis was monitored using a pH meter (pH Meter NineFocus NF2000 UltraLowVol, Mettler-Toledo International Inc., Columbus, OH, USA). The studied preparations, DDAC, FAEO8, and DDAC-FAEO8, did not contain potassium ions in their composition; therefore, they did not affect the accuracy of the analysis results.

The samples for analysis were taken at 5-minute intervals during the 1st h of incubation. Then, sampling occurred once every 30 min. The samples were filtered through a cellulose nitrate membrane filter (pore size 0.2 μm). The concentration of K+ in the samples was determined using a PerkinElmer Analyst 400 atomic absorption spectrometer (PerkinElmer, Waltham, MA, USA) in flame emission mode (wavelength: 766.5 nm; slit height: 0.7 nm; air-acetylene flame). Standard potassium (Merck, Darmstadt, Germany) was used for the calibration curve (0.1-10 ppm). The sensitivity limit is 0.01 ppm [51, 61].

### Fluorometric measuring of microbial cells’ membrane potential with DiSC3(5)

The membrane potential of *S. aureus*, *E. coli*, and *S*. Typhimurium cells was measured as described in detail by Winkel *et al*. [62]. Daily bacterial cultures containing 10^9^ CFU/mL were diluted with sterile MPB to an OD_600_ of 0.3. Next, 150 μL of suspension was transferred into 96-well light-proof polystyrene plates for fluorometric studies (Deltalab, Barcelona, Spain). After obtaining background fluorescence values, DiSC3(5) dye dissolved in DMSO was added to each well to a final concentration of 1 μM. Fluorescence quenching was measured until a stable signal intensity was achieved, after which the test compounds were added at a concentration of 2× MIC or 0.1 μM valinomycin (positive control). Negative control samples were not treated with any preparations or valinomycin. Fluorescence was detected using a fluorimeter (Tecan, Mannedorf, Switzerland) every 5 min (λexcitation – 610 nm, λemission – 665 nm) for 60 min [62].

### Toxicology studies

Eight-week-old and weighing 200 ± 20 g Wistar male rats, as well as five-month-old female New Zealand White rabbits weighing 3,500 ± 350 g, were used for the study, obtained from the Scientific Center for Biomedical Technologies at the Stolbovaya Branch of the Federal Medical Biological Agency in Russia. Passing the veterinary inspection was confirmed by an electronic health certificate issued through the Russian Federal State Information System “Mercury”. The animals were quarantined for 14 days before the experiment began. The rats had free access to water and commercial pelleted feed *ad libitum*. They were housed under standard conditions, including a 12-h light/12-h dark cycle, in a temperature-controlled environment (22°C ± 1°C) with a relative humidity of 55 ± 5%.

To monitor animal welfare, daily observations were conducted. All results were recorded in an approved protocol, which included the following items: appearance, weight loss, coat condition, physiological functions, respiration assessment, cage assessment, animal behavior (tension, flinching when handled), motor activity (posture, gait, mobility), and experimentally induced changes (tumors, wounds, scratches). The total score was calculated based on a combination of indicators. The animals were included in the group by the method of random sampling according to the criteria – age and body weight (±10% of the average weight). The criteria were established a priori. The observers were blinded to groups in all studies [63].

The toxicological evaluation of the complex disinfectant DDAC-FAEO8 at a concentration of 40 mg/mL was carried out. The toxicity of the disinfectant was classified in accordance with the requirements of Interstate standard 32419-2022 “Chemical hazard classification. General requirements”, 2022 and Globally Harmonized System of Classification and Labeling of Chemicals (GHS Rev. 10, 2023).

### Acute toxicity study

The study was conducted in accordance with OECD guideline for testing of chemicals No. 423, 2001 and Guidelines 1.2.1105-02 “Assessment of toxicity and hazard of disinfectants”, 2002. Rats were weighed and then divided into three groups, with six individuals in each. Aqueous solution of DDAC-FAEO8 was administered to the rats orally in doses of 50, 300, and 2000 mg/kg of body weight. The control group received only drinking water. The water solution of preparation was administered once in the morning, on an empty stomach, through a feeding tube in a volume of 0.05 mL/100 g to 0.2 mL/100 g. The last feeding was in the evening before the experiment day, while the drinking regime was maintained. Individuals in the control group received water in the maximum volume (0.2 mL per 100 g of weight).

The animals were monitored for two weeks after the administration of the substance. The rats’ general condition and weight, appetite, preservation of motor functions, coordination of movements, the presence or absence of seizures, response to sound stimuli, the condition of the mucous membranes and coat, the type and consistency of feces, etc., were taken into account. On day 14 after the administration of the disinfectant, the rats were euthanized humanely using CO_2_ asphyxiation. Necropsy was performed, and the condition of internal organs and tissues was assessed [64].

### Sub-acute toxicity study

In light of the fact that the maximum tolerated dose was more than 2000 mg/kg, the method recommended for low toxicity compounds was used (Interstate standard “Determination of toxicity by repeated/multiple oral administration in rodents. 28-day test”, 2014 and OECD guidelines No. 407, 2008). The preparation was administered to rats for 28 days as an aqueous solution at a dose of 1000 mg/kg. The statistical groups comprised ten individuals each. Both mortality and functional accumulation were considered. A range of parameters was evaluated. To determine the state of the central nervous system, a summation threshold index (STI) was used. For this purpose, the STI-01-M device (ProfLab, St. Petersburg, Russia) was utilized. Neuromuscular excitability of animals was determined using electrodes by contraction of interdigital muscles with increasing current supply. The animal was fixed and placed with its hind legs on an electrode moistened with saline solution. When the animal calmed down, weak electric current pulses (pulse frequency 0.5 Hz, interval between pulses 0.5 sec) were applied at increasing voltages until the interdigital muscles contracted. The result was taken into account in the points shown by the device at the moment of stopping. The measurements were carried out in triplicate with an interval of at least 10 min. The stability of this indicator in animals was taken into account when selecting them for the experiment [65].

Blood samples were collected from the tail vein into heparinized tubes and analyzed for red blood cell count, white blood cell count, and hemoglobin. The analysis was conducted using a Medonic CA-620 BALDER hematology analyzer (Boule Medical AB, Sweden), employing Uni-Gem reagents (Reamed, Russia). To obtain serum for biochemical analysis, blood was collected in vacuum tubes and centrifuged at 946 × *g* for 15 min at 25°C in a centrifuge (DM0506, DLab Scientific, Shenzhen, China). The impact of DDAC-FAEO8 on liver function was assessed by measuring serum total protein concentration using an IRF-22 refractometer (Tagler, Moscow, Russia). A spectrophotometer PE5400UF (Ekroskhim, St. Petersburg, Russia) was used to determine the amount of SH groups in blood serum according to the method [66]. The functional state of the kidneys was studied by measuring diuresis, urine specific gravity, protein and chloride content in the urine [67]. The immunoglobulin levels were determined using the turbidometric method [68]. The temperature was measured rectally using a thermometer (BIO-IRB153; LiderMed Group, Moscow, Russia) for small rodents. The animals were weighed on high-precision scales, Gosmetr VPT-12 (Gosmetr, St. Petersburg, Russia).

### Acute dermal irritation/corrosion testing

The study was conducted in accordance with the OECD Guideline for the Testing of Chemicals No. 404 “Acute Skin Irritation/Corrosion”, 2015. For a preliminary assessment of irritant and corrosive effects, two healthy rabbits with intact skin were used. The animals were treated with a concentrated complex preparation containing 500 mg/mL DDAC and 87.6 mg/mL FAEO8, as well as a diluted disinfectant solution at concentrations of 40 mg/mL and 7.0 mg/mL, respectively. The exposure time was 3 min. If no serious skin reactions were observed at this stage, exposure periods of 1 and 4 h were tested. 0.5 mL of ddH_2_O was applied to the control areas. At least 24 h prior to treatment, the animals had their hair trimmed on an inaccessible dorsal area of the trunk measuring approximately 10 × 8 cm. The procedure was performed carefully, avoiding damage to the skin. The solutions in 0.5 mL volumes were applied to gauze and secured with a non-irritating patch over a 6 cm² area of skin. To prevent excessive exposure, the dressings were removed after the indicated exposure periods. The skin was thoroughly washed with tap water at 22°C ± 2°C and blotted dry with a clean cloth. After removing the dressings, the test areas were inspected and assessed for signs of erythema and edema, as well as local and systemic toxic side effects. The results were recorded at 1, 4, 24, 48, and 72 h, as well as at 14 days after the dressings were removed. The scoring scale of dermal irritation/corrosion signs is presented in OECD 404 [69].

Based on the preliminary assessment results, a group of three animals was formed. Water was applied to the left side of each animal (control), while the right side was treated with a diluted disinfectant solution for 1 h. The design of the control test was similar to the preliminary one.

### Statistical analysis

The data analysis was performed using GraphPad Prism 10.4.0.621 (GraphPad, San Diego, CA, USA). Normal distribution of the dataset was confirmed using the Shapiro-Wilk test. One-way analysis of variance with multiple comparisons was used to identify statistically significant differences between groups. The Student’s t-test and Tukey’s test were used to determine the significance of differences at a threshold of p < 0.05.

All measurements were performed in triplicate. Results are presented as mean (M) and SD (for microbiological studies), as well as standard error of the mean (±SEM) (for toxicological studies).

## RESULTS

### MICs determination and FICI analysis results

During the initial phase of microbiological analysis, MICs were determined to assess the antimicrobial efficacy of DDAC, FAEO8, and a 40:7 (v/v) mixture of DDAC and FAEO8. The selection of this particular surfactant-to-disinfectant ratio was based on the findings of our preliminary investigations into FAEO8’s cleaning capabilities across diverse dilutions. The stability of the DDAC concentration in the disinfectant solution, as well as the absence of phase separation among the components of the preparation over 1 year at pH values from 4 to 9, was confirmed. Table 2 summarizes the MIC results obtained.

**Table 2:** Minimum inhibitory concentration values (µg/mL).

Preparations	*Staphylococcus aureus*	*Escherichia coli*	*Salmonella* Typhimurium
DDAC	0.45 ± 0.02	1.30 ± 0.06	1.50 ± 0.07
DDAC-FAEO8	0.25 ± 0.01	0.90 ± 0.03	1.10 ± 0.05
FAEO8	> 125	> 125	> 125

p < 0.05.

The data obtained showed that, compared with free DDAC, the inhibitory activity of DDAC-FAEO8 increased by 44.4%, 30.8%, and 26.7% against *S. aureus*, *E. coli*, and *S*. Typhimurium, respectively. The effect of DDAC and the complex preparation on Gram-negative bacteria was 2.9-3.3 and 3.6-4.4 times weaker than on Gram-positive microorganisms. Notably, no antimicrobial activity was observed for the surfactant at an equivalent concentration. The FICI values were 0.56, 0.69, and 0.73 for DDAC-FAEO8 against *S. aureus*, *E. coli*, and *S*. Typhimurium, respectively. Therefore, an additive effect can be assumed.

### Survival of microorganisms under preparation treatment

**Figure 2:**
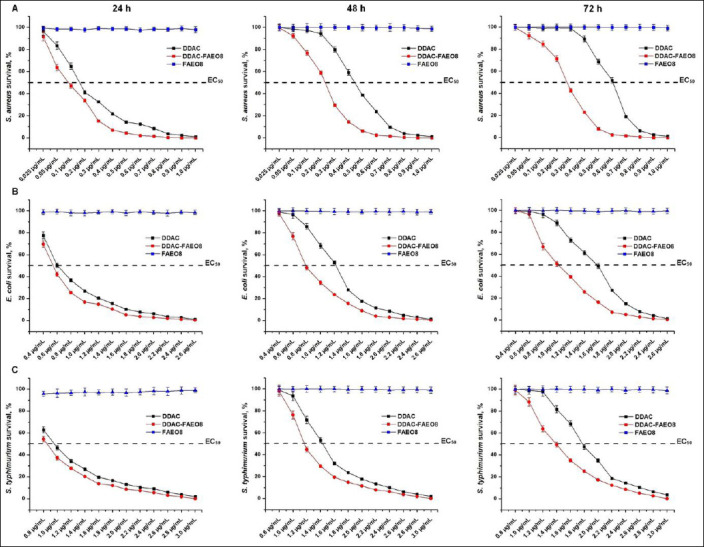
Survival of (A) *Staphylococcus aureus*, (B) *Escherichia coli*, and (C) *Salmonella* Typhimurium under preparation treatment for 24, 48, and 72 h. p < 0.05.

To determine the bactericidal effect of the preparations, the percentage of bacterial survival was calculated at different dosages (Figure 2).

As follows from the data presented, after 24 h, the sub-MICs of DDAC and DDAC-FAEO8 destroyed no more than 73.05–79.56% and 62.71–76.24% of the studied microorganisms, respectively. However, the MIC of the complex preparation was 1.36–1.8 times lower than that of free DDAC. The use of DDAC doses ≥2×MIC allowed achieving 97.82-99.14% inhibition of staphylococcal growth, while in combination with FAEO, 99.96–99.99%. The effective dose of DDAC-FAEO8, which inhibited 99.65% of *E. coli* and 99.99% of *S*. Typhimurium, was also 2× the MIC. Free DDAC at a similar concentration inhibited 98.89% and 98.05% of bacteria, respectively. After 48 and 72 h, bacterial growth was absent when exposed to ≥2× MIC of both free DDAC and DDAC-FAEO8. In contrast, <2× MIC-treated samples showed increased growth as the preparation concentrations reduced. Remarkably, the surfactant was found to be non-bactericidal at all concentrations tested for up to 72 h.

In addition, the 50% effective concentration (EC50) values were established. For example, 72 h after treatment with *S. aureus*, *E. coli*, and *S*. Typhimurium, the EC50 of free DDAC was 0.62, 1.58, and 1.71 μg/mL, respectively. By contrast, for DDAC-FAEO8, these values were 0.27, 1.03, and 1.39 μg/mL.

### Results of IZDs evaluation

**Figure 3:**
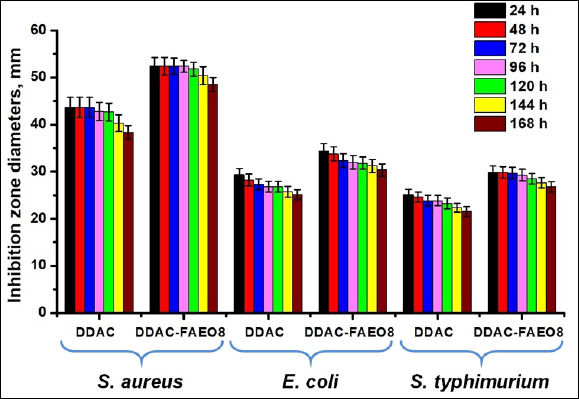
Inhibition effect of Didecyldimethylammonium chloride and its complex with fatty alcohol ethoxylate on *Staphylococcus aureus*, *Escherichia coli*, and *Salmonella* Typhimurium. p < 0.05.

The antimicrobial effect of the preparations in concentrations that suppress more than 99% of bacteria was assessed on a solid nutrient medium for 168 h (Figure 3).

It is evident that the inhibition zones of microorganisms diminished over time. Following 7 days of treatment with DDAC, the inhibitory zones of *S. aureus*, *E. coli*, and *S*. Typhimurium decreased by 12.12%, 14.38%, and 14.17%, respectively. When employing DDAC-FAEO8, the decrease was 7.4%, 11.57%, and 10.40%. Furthermore, compared with free DDAC, the combined preparation increased the average inhibition zone diameter by 22.13% for *S. aureus*, 19.27% for *E. coli*, and 22.43% for *S*. Typhimurium.

It should be noted that there was no noticeable reduction in the growth inhibition zones of staphylococci for 4 days when treated with DDAC, and for 5 days when exposed to the FAEO8-containing preparation, whereas for *E. coli* and *S*. Typhimurium, these changes began on the 2nd day. In the surfactant experiment, continuous bacterial growth was observed across the entire surface of the Petri dishes.

### Bacterial cells SEM results

**Figure 4:**
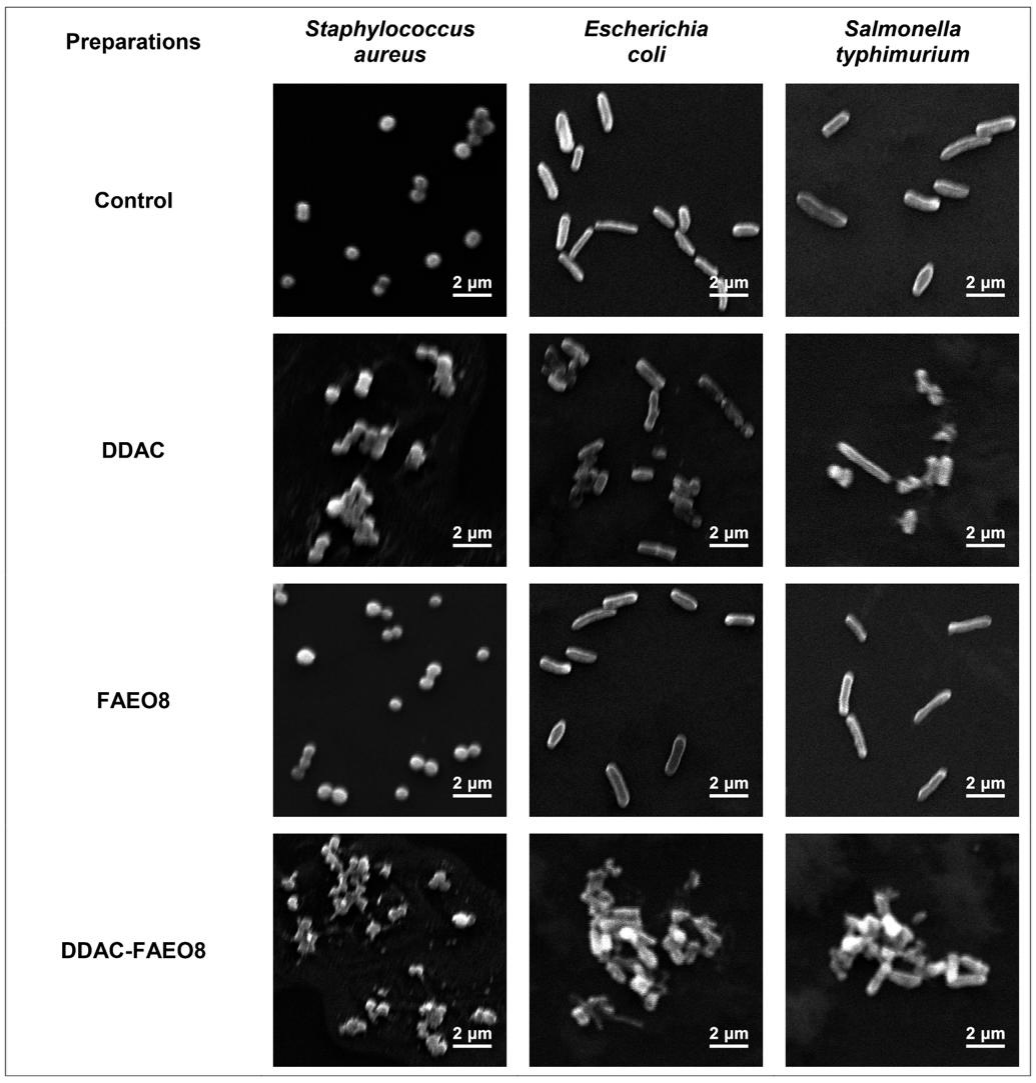
Scanning electron microscopy images of *Staphylococcus aureus*, *Escherichia coli*, and *Salmonella* Typhimurium incubated with preparations (4000× magnification).

SEM was used to study the morphological changes in *S. aureus*, *E. coli*, and *S*. Typhimurium cells caused by DDAC, FAEO8, and complex preparation (Figure 4).

The control images showed clear edges, a characteristic size and shape of bacterial cells. *S. aureus*, *E. coli*, and *S*. Typhimurium cells exposed to 2×MIC of DDAC deformed and aggregated, forming conglomerates. However, the Gram-negative bacteria appeared less damaged. Treatment with the same concentration of complex preparation resulted in lysis of cell walls and complete destruction of bacteria. Hence, destructive processes were more pronounced. The microorganisms incubated with surface-active agents appeared visually undamaged and were comparable to control samples.

The percentage of lysed staphylococcal cells was 95.28%, *E. coli* – 96.52%, and *S*. Typhimurium – 95.74%.

### K+ leakage evaluation

**Figure 5:**
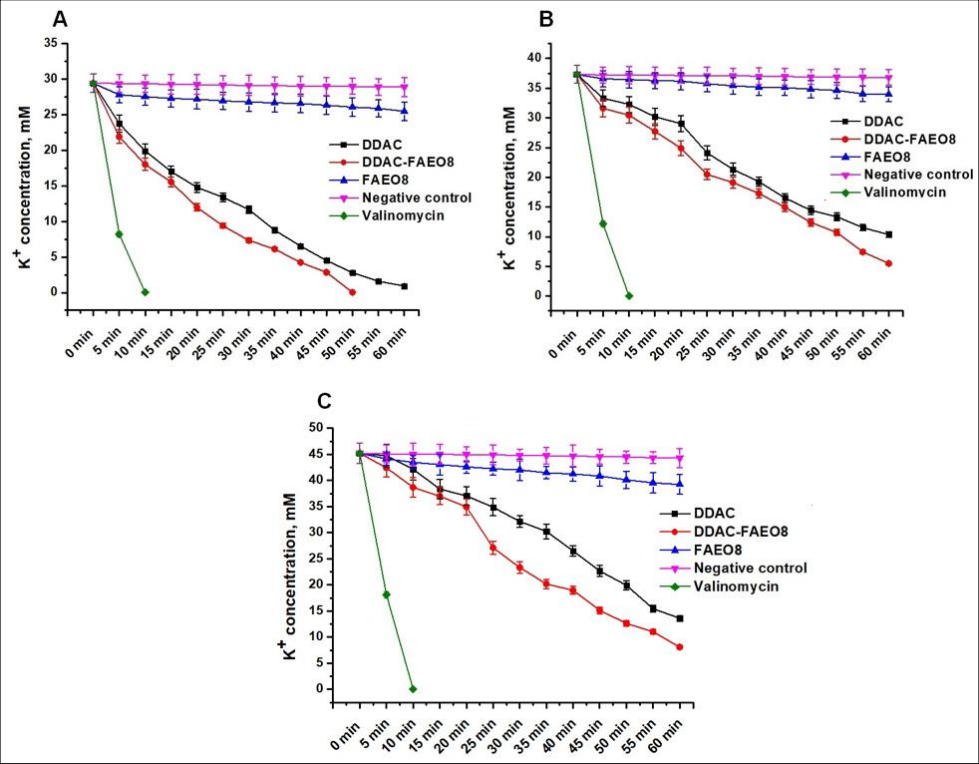
Leakage of potassium ions from (A) *Staphylococcus aureus*, (B) *Escherichia coli*, and (C) *Salmonella* Typhimurium cells. p < 0.05.

It was found that the potassium ion concentrations in the control samples of *S. aureus*, *E. coli*, and *S*. Typhimurium were 29.43 ± 1.29, 37.32 ± 1.51, and 45.19 ± 1.97 mM, respectively. The K+ efflux profiles from cells treated with preparations at a concentration of 2× MIC over time are shown in Figure 5.

A strong potassium ion leakage was observed from *S. aureus*, *E. coli*, and *S*. Typhimurium cells attacked by both DDAC and DDAC-FAEO8. However, treatment of staphylococcal cells with the complex preparation resulted in a near-complete release of K+ (99.9%) within 50 min, whereas free DDAC induced the efflux of 96.8% of potassium ions within 60 min. The leakage of 99.9% of K+ under the influence of DDAC occurred only 70 min after the start of observations.

K+ leakage from *E. coli* and *S*. Typhimurium was less pronounced, reaching 72.3% and 69.88% after DDAC exposure for 60 min, while DDAC-FAEO8 treatment released 85.3% and 82.0%, respectively, over the same time. Further analysis showed that a release of ~100% K+ was observed after 2 and 1.5 h, respectively.

Interestingly, incubation of bacterial cells with FAEO8, which did not exhibit antimicrobial activity, resulted in leakage of 13.42% potassium from *S. aureus* cells, 9.03% from *E. coli*, and 13.19% from *S*. Typhimurium. In this regard, we recorded potassium ion leakage for an additional 9 h. As a result, over the 10-h experiment, a total of 19.98%, 17.55%, and 16.89% of the K+ leaked from the treated *S. aureus*, *E. coli*, and *S*. Typhimurium cells, respectively. However, it was noted that at the 5th h of observation, this process slowed further, and at the 8^th^ h, it stopped completely.

It should be noted that no changes in K+ leakage were detected in DDAC and DDAC-FAEO8-treated samples after 10 h of recording.

K+ leakage from negative control cells was observed but did not exceed 2% over 10 h. As expected [70], treatment of cells with the ionophore valinomycin resulted in rapid K+ release from microbial cells, with almost complete efflux occurring within 10 min.

### Analysis of microbial cells’ membrane potential

**Figure 6:**
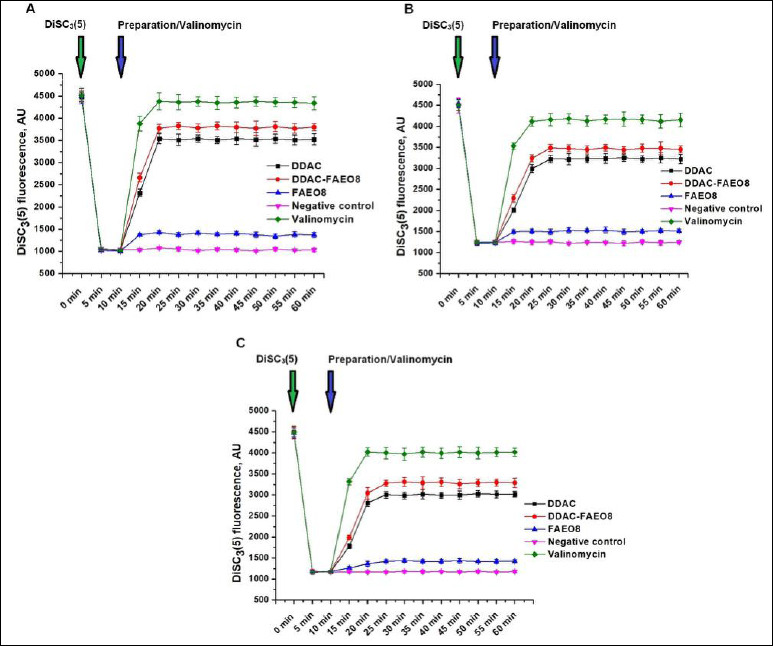
Fluorescence intensity changes of DiSC3(5) in (A) *Staphylococcus aureus*, (B) *Escherichia coli*, and (C) *Salmonella* Typhimurium cells. The time points of dye and preparations or valinomycin addition are highlighted with green and blue arrows, respectively. p < 0.05.

This study aimed to identify changes in cell membrane potential in response to the tested preparations. The results are shown in Figure 6.

It is evident that the accumulation of the cationic dye DiSC3(5) within microbial cells led to fluorescence quenching. However, the addition of DDAC, DDAC-FAEO8, or valinomycin (positive control) induced rapid dye release, attributable to membrane depolarization. When cells were treated with valinomycin, the percentage of dye leaked out at the first plateau point was 96.7% for staphylococci, 88.2% for *E. coli*, and 85.6% for *S*. Typhimurium. This figure was 11.3% for FAEO8-treated *S. aureus*, while for *E. coli* and *S*. Typhimurium under the same conditions, it was 8.1% and 7.4%, respectively. The free DDAC influence resulted in 72.2% DiSC3(5) release from staphylococcal cells, but only 60.7% and 55.1% from *E. coli* and *Salmonella* cells, respectively. The complex preparation removed 79.0% of the dye from *S. aureus* cells, but a lower percentage from Gram-negative microorganisms – 68.8% and 63.2%, respectively. The absence of DiSC3(5) spontaneous release from microbial cells during the measurement was confirmed in the negative control (untreated cells).

### Acute toxicity assessment of DDAC-FAEO8

As a result of a previous investigation into the disinfectant properties of DDAC-FAEO8, it was established that its maximum bactericidal concentration for veterinary surveillance facilities was 40 mg/mL. Hence, a toxicological assessment at this concentration was conducted in subsequent experiments.

Oral administration of the preparation’s aqueous solution at doses of 50, 300, and 2000 mg/kg to rats did not result in death. Therefore, a dose of 2000 mg/kg can be considered the maximum tolerated. No clinical manifestations of intoxication were noted in the animals. Following administration of the preparation, no differences were observed between the treated individuals and the control group. No pathological changes were detected during the post-mortem examination.

The body weight of rats did not differ significantly from the weight of control animals 7 and 14 days after a single administration of the disinfectant at the indicated doses (Table 3).

**Table 3:** The rats’ body weight after a single oral administration of didecyldimethylammonium chloride complexed with fatty alcohol ethoxylate.

Doses, mg/kg	Body weight, g (Before the administration)	Body weight, g (7 days)	Body weight, g (14 days)
50	190.8 ± 1.9	201.3 ± 2.1	210.5 ± 2.5
300	192.3 ± 1.7	205.3 ± 2.3	212.7 ± 2.2
2000	193.6 ± 2.1	205.2 ± 2.8	211.9 ± 2.5
Control	193.5 ± 1.8	207.5 ± 2.5	213.5 ± 2.1

p < 0.05.

Due to the lack of animal mortality, it has not been possible to determine the toxicity parameters of LD0, LD50 and LD100. Given that the LD50 of DDAC-FAEO8 after a single oral administration exceeds 2000 mg/kg body weight, this disinfectant can be classified as a substance with low toxicity, belonging to category V.

### Sub-acute toxicity assessment of DDAC-FAEO8

During the investigation of the sub-acute toxicity of this preparation, no animal deaths were recorded throughout the entire experimental period. The rats remained active, agile, and exhibited normal feeding behavior, suggesting that the compound does not accumulate in the body. The results of the animals’ examination to identify functional accumulation are presented in Table 4.

**Table 4:** Functional indices of rats’ condition in a sub-acute experiment.

Indices	Days	Groups (Control)	Groups (Treated)
Body weight, g	0	209.2 ± 6.0	208.1 ± 6.3
	7	227.3 ± 6.3	221.6 ± 7.0
	14	245.2 ± 7.3	237.8 ± 7.1
	28	272.2 ± 11.2	254.7 ± 11.5
Immunoglobulin, mg/kg	14	14.2 ± 0.8	13.6 ± 0.6
	28	14.3 ± 1.3	13.5 ± 0.5
Rectal temperature, °C	14	37.3 ± 1.1	37.4 ± 1.8
	28	37.1 ± 2.1	37.6 ± 2.1
Summation threshold index (STI), conventional units	14	4.6 ± 0.5	5.5 ± 0.7
	28	4.2 ± 0.7	5.7 ± 0.6
Hemoglobin, g/L	14	14.7 ± 0.1	15.3 ± 0.3
	28	15.6 ± 0.2	15.7 ± 0.4
Leukocytes, ×10⁹/L	14	8.11 ± 0.37	7.38 ± 0.49
	28	8.20 ± 0.37	7.47 ± 0.49
Red blood cells, ×10¹²/L	14	6.38 ± 0.21	6.42 ± 0.25
	28	6.50 ± 0.31	6.40 ± 0.30
Daily diuresis, mL	14	5.21 ± 0.30	8.83 ± 0.31*
	28	5.05 ± 0.61	9.31 ± 0.59*
Specific gravity of urine, g	14	1.011 ± 0.006	1.010 ± 0.005
	28	1.012 ± 0.006	1.011 ± 0.005
Proteinuria, g/L	14	0.50 ± 0.02	0.51 ± 0.02
	28	0.51 ± 0.01	0.49 ± 0.02
Chloride-urine test, mg/mL	14	3.81 ± 0.21	3.69 ± 0.25
	28	3.94 ± 0.25	3.73 ± 0.28
Hippuric acid in urine during benzoic acid loading, mg/mL	14	18.2 ± 2.3	18.4 ± 2.2
	28	17.4 ± 2.3	18.6 ± 2.1
Total sulfhydryl (SH)-groups in serum, µM/100 mL	14	—	—
	28	26.1 ± 1.2	25.9 ± 2.3
Total serum protein, g/%	14	—	—
	28	7.37 ± 0.13	7.48 ± 0.51

* p < 0.05.

A reduction in body weight was observed in animals receiving DDAC-FAEO8 when weighed on days 7, 14, and 28. The examination of rats at 14 and 28 days post-experiment revealed increased diuresis.

No treatment-related macroscopic abnormalities were observed in rats at necropsy compared with the control group. The stomach was normal in size and shape and contained chyme. The gastric mucosa was folded, pink, and glossy, with no visible lesions or signs of irritation. The liver was normal in size and morphology. The capsular surface was smooth and homogeneous, dark red. The tissue on the cut was of homogeneous consistency and moderately dense. The pancreas was flat, pale pink, lobulated, and moderately dense, without focal lesions. The spleen was normal in size and shape, dark cherry in color, with a smooth surface and thin capsule. The cut surface revealed small grayish lymphoid follicles. The heart was normal in size and configuration. No gross evidence of edema or dilation was observed. Vascular filling of small veins and intermuscular capillaries was within normal limits. The kidneys did not change in size and shape and had a smooth brownish surface. The capsule was thin, transparent, and easily removed. The cut surface showed preserved corticomedullary differentiation. The adrenal glands were pale yellow, rounded, and moderately dense, with no visible abnormalities. The urinary bladder contained clear urine. The mucosa was smooth, pale, and glistening. The thymus was predominantly located within the thoracic cavity and was normal in size, color, and lobular architecture. The brain completely occupied the cranial cavity and was elastic in consistency. The cut surface demonstrated a clear demarcation between gray and white matter. No gross pathological findings were identified.

### Acute dermal irritation/corrosion test results

Dermal irritation and corrosion effects were evaluated *in vivo* after topical application of the tested solutions of the complex preparation to rabbit skin. The grading of skin reactions is presented in Tables 5 and 6.

**Table 5:** Erythema and edema formation after didecyldimethylammonium chloride complexed with fatty alcohol ethoxylate solutions treatment (preliminary test).

Observation time	Erythema (DDAC concentration, mg/mL: 40)	Erythema (500)	Edema (40)	Edema (500)	Total score (40)	Total score (500)
Before applying solutions	0	0	0	0	0	0
Solution exposure*						
3 min	0	2	0	1	0	3
1 h	1	×	0	×	1	×
4 h	×	×	×	×	×	×
After treatment						
1 h	1	3	0	2	1	5
4 h	0	3	0	3	0	6
24 h	0	3	0	2	0	5
48 h	0	3	0	2	0	5
72 h	0	3	0	2	0	5
14 days	0	0	0	0	0	0

× – No treatment.

* The results were assessed immediately after solution removal.

**Table 6:** Erythema and edema formation after 1-h treatment with DDAC-FAEO8 at a concentration of 40 mg/mL.

Observation time	Erythema (Animals # 1,2,3)	Erythema (Average score)	Edema (Animals # 1,2,3)	Edema (Average score)	Total score (Average)
Before applying solutions	0 0 0	0	0 0 0	0	0
Solution exposure* 1 h	1 1 1	1	0 0 0	0	1
After treatment					
1 h	1 1 1	1	0 0 0	0	1
4 h	0 0 0	0	0 0 0	0	0
24 h	0 0 0	0	0 0 0	0	0
48 h	0 0 0	0	0 0 0	0	0
72 h	0 0 0	0	0 0 0	0	0
14 days	0 0 0	0	0 0 0	0	0

* The results were assessed immediately after solution removal.

An assessment of skin irritation caused by concentrated DDAC in a preliminary experiment showed the formation of moderately pronounced pinkish-red erythema (2 scores) and slight edema (1 score) after 3 min. Severe rabbit anxiety and attempts to remove the dressing were noted. Despite the animal calmed down after the solution was removed, discomfort signs were observed for 24 h. Hence, the increase in exposure was deemed inhumane.

One hour after the experiment, edema increased to mild (2 scores) and erythema to 3 scores. After 4 h, the edema was assessed at 3 scores. 24-h observation revealed a decrease in edema to 2 scores, unchanged erythema, and the formation of thin scabs. The test areas persisted in a similar state after 48 and 72 h. At the end of the observation period, redness and swelling were absent; the treated site was covered with hair.

When the preparation was applied at a diluted concentration, no changes were detected after 3 min, whereas 1-h testing showed no edema and mild pink erythema (1 score), providing grounds to terminate the experiment. There were no further changes in the area with a 3-minute exposure throughout the entire observation period.

One hour after the end of treatment, mild erythema (1 score) without signs of edema was observed in the area following 1 h of exposure to the solution. After 4 h, erythema and edema had completely disappeared. The experimental areas did not differ from the control ones. No changes were detected during further monitoring. It is important to note that no local or systemic toxic effects were observed at either concentration.

The analysis of the preliminary test results provided the basis for selecting a concentration of 40 mg/mL and a 1 h exposure for the control test (Table 6).

As can be seen, the results of the control experiment were identical to those of the preliminary experiment. No significant differences in response were observed between individuals.

## DISCUSSION

### Rationale for the selection of chemical components and microorganisms

DDAC is a broad-spectrum biocide with antimicrobial activity, widely employed as a disinfectant in both human and veterinary medicine. Nonetheless, enhancing the antimicrobial efficacy of the preparation while maintaining its safety remains a critical challenge. Over the years, various approaches have been used to achieve this objective. For example, as mentioned earlier, the use of certain surface-active agents can increase the bioavailability of molecules. However, there is still no data on fatty alcohol ethoxylates, particularly FAEO8. Therefore, this work is devoted to studying this issue.

The antimicrobial properties of DDAC, FAEO8, and the resulting complex were assessed against opportunistic strains of *S. aureus*, *E. coli*, and *S*. Typhimurium, which are often used as test models for various microbiological experiments [71-76]. Moreover, staphylococcosis, escherichiosis, and salmonellosis pose a serious threat to animal health, food safety, and public health [77–83].

### Microbiological evaluation

*E. coli* and *S*. Typhimurium showed greater resistance to DDAC and DDAC-FAEO8 than *S. aureus* (Table 2). As is known, Gram-negative microorganisms, unlike Gram-positive bacteria, have a complex outer membrane, including a layer of lipopolysaccharides, a lipid bilayer and transmembrane proteins [84–86]. This structure results in low permeability of antibiotic molecules of various groups [87–89].

The obtained MIC values for DDAC were comparable with the results of Ioannou *et al*. [90], Jansen *et al*. [34], Yoshimatsu *et al*. [91] and Buffet-Bataillon *et al*. [16]. According to some researchers, the MIC of preparations and their minimum bactericidal concentration (MBC) are not always the same; most often, the MBC is higher than the MIC. Therefore, to achieve a bactericidal or persistent bacteriostatic effect, different MIC multiplicity may be required [92–96]. This trend can also be seen in our work. The results obtained from the survival experiments indicated that the MIC did not fully suppress the microbial population (Figure 2). Hence, we selected a DDAC concentration that would provide a long-lasting bacteriostatic effect (Figure 3). There is evidence that some alcohol ethoxylates can destabilize the bacterial cell wall, leading to leakage of intracellular fluid [51]. The FAEO8 used in our study did not exhibit intrinsic inhibition, as demonstrated in survival experiments and by measuring the diameters of bacterial growth zones (Figures 2 and 3). Interestingly, despite the surfactant’s lack of inhibitory properties, its addition to DDAC clearly enhanced antimicrobial potential (Table 2, Figures 2 and 3). To explain this effect, SEM of microorganisms was performed (Figure 4). Treatment of bacteria with surfactant alone did not result in visible cell wall damage, whereas DDAC and DDAC-FAEO8 caused cell deformation. Although both cases demonstrated disruption of cell wall integrity, the destructive effects were more pronounced in samples treated with the complex preparation. These findings may indirectly indicate that FAEO8 changes the permeability of the bacterial cell wall, not through mechanical destabilization, but rather through a change in its chemical composition or a disruption of the cell’s transmembrane potential. It is already known that some antibiotics [97–99] and some non-ionic surfactants [100, 101] have similar mechanisms. However, we have not found any literary sources describing such an effect in fatty alcohol ethoxylates. The closest study is the work of Moore *et al*. [51].

To study the permeability of treated bacterial cells, the leakage of potassium ions (K+) from the intracellular space was evaluated, since this approach is recognized as a reliable indicator of damage to the bacterial cell wall [61, 102–104]. The results demonstrated active K+ leakage from DDAC- and DDAC-FAEO8-treated bacterial cells, likely due to the formation of unregulated channels (Figure 5). The observed effect for DDAC was previously described in the work of Ioannou *et al*. [90], which showed the efflux of potassium ions (from 10 to 90% depending on the biocide’s concentration) from *S. aureus* cells already in the first five minutes of the experiment. The addition of fatty alcohol ethoxylate to DDAC clearly increased the intensity of this process. K+ leakage, although not as active, induced by free FAEO8 was detected. A temporary change in the membrane potential of the cell wall can be assumed, since the leakage process stopped at 8 h of observation. At the same time, the cell’s ability to divide was apparently not impaired, since no structural damage to the cell wall was observed (Figure 4). This is also confirmed by the absence of visible changes in bacterial colony growth (Figure 3). The cessation of K+ efflux from cells can be explained by the activation of repair and remodeling systems that repair damage to the microbial wall and ensure sealing of the cell’s internal space. For instance, these processes are described in detail in the work of Bramkamp and Scheffers [105]. The effect of fatty alcohol ethoxylates on bacterial cells that we discovered is very interesting and requires detailed study, which will be the subject of our further research. K+ leakage from negative control cells did not exceed 2% during the entire observation period. Presumably, this effect may be a consequence of the cell collection and washing procedure, as noted in studies by Moore *et al*. [51] and Korobov *et al*. [61].

Membrane potential is one of the factors influencing the viability of bacterial cells [106, 107]. To confirm the responsibility of the studied preparations for their change, the voltage-sensitive fluorescent dye DiSC3(5) was used, which is able to penetrate the cell wall and accumulate in polarized microbial cells (self-quenching of fluorescence). However, upon depolarization of the cell membrane, the dye is rapidly released from the cells, leading to dequenching [62, 106, 108-111]. We observed a similar process in our work during the fluorimetric measurement of the membrane potential of *S. aureus*, *E. coli*, and *S*. Typhimurium cells after adding the tested preparations to them (Figure 6). Rapid dye release was induced by both the DDAC-FAEO8 and free DDAC. Interestingly, when FAEO8 was added, an increase in the DiSC3(5) fluorescence signal was also observed, albeit less pronounced. It confirms the previously stated assumption that surfactants can alter the membrane potential of microbial cells.

### Toxicology testing

An evaluation of acute and sub-acute toxicity, as well as an acute dermal irritation/corrosion test, is a necessary step before proceeding to other toxicological tests [112, 113]. The choice of the dose range for the acute study of the complex preparation was based on the toxicity data of the included compounds. According to the GHS, DDAC is classified in toxicity category III [114]. FAEO8 (Lutensol AO8) is declared by the manufacturer as a compound with low toxicity [115]. In an acute toxicology study, single administration of the DDAC-FAEO8 at doses of 50, 300, and 2000 mg/kg did not result in a decrease in rat weight (Table 3), nor in intoxication or death. When the disinfectant was administered again at a dose of 1000 mg/kg for 28 days, no fatal outcomes were observed. The minor physiological changes were noted (Table 4). The observed increase in diuresis, on the one hand, may indicate kidney pathology, given the increased renal blood flow. However, because the other parameters in experimental animals did not differ significantly from control values, it is reasonable to assume that, in this case, the increase in diuresis is a nonspecific symptom (physiological polyuria) that does not indicate a specific disease. It appears that under prolonged administration, the disinfectant can cause increased thirst, leading to excess fluid intake (which was indeed noted) and subsequently increasing diuresis volume. Since oral administration of this preparation is not intended, there is no risk associated with this for animals. Thus, the administration of the preparation at the maximum dose did not result in animal death. Minor changes were noted, including increased water consumption, enhanced diuresis, and a slight reduction in body weight. However, this decrease in weight was not statistically significant compared to the control group.

Since no macroscopic changes were detected over necropsy compared to the control group, it was considered appropriate to conduct histological studies during chronic oral administration in accordance with OECD Test N 408:2018 “Guideline for Testing of Chemicals. Repeated-dose 90-day oral toxicity study in rodents”.

Due to the lack of a median lethal dose under both single-dose and repeated-dose conditions, it was not possible to determine the level of disinfectant accumulation. All observed changes occurred after multiple administrations of DDAC-FAEO8 at a cumulative dose of 28 g/kg. Therefore, the true risk associated with the preparation is only potential, provided that proper safety measures are not implemented and the recommended dose is significantly exceeded.

Concentrated DDAC solution (50 and 80% v/v) is known to cause a corrosive reaction within 3 min of skin exposure. Therefore, the disinfectant is classified as corrosive and skin-irritating (category 1B) [37, 116]. We observed a comparable effect when applying DDAC to rabbits’ skin in our study (Table 5). In contrast, the treatment with a diluted solution caused only slight erythema without edema, which completely disappeared within 4 h (Tables 5 and 6). It can be assumed that the skin irritation caused by DDAC was dose-dependent. A similar observation was reported by Jann *et al*. [117]. Hence, the diluted solution of the complex preparation cannot be categorized as a skin irritant (according to the Globally Harmonized System of Classification and Labeling of Chemicals).

Direct contact with concentrations of FAEO8 (Lutensol AO8) from 60 to 100% may cause redness, irritation, or defatting of the skin [115]. Since its content in the tested solutions was significantly lower, the surfactant’s contribution to the observed skin damage appears insignificant.

Overall, the negative impact of the complex preparation on the skin is limited by the scenario of its use, which involves disinfection of veterinary surveillance facilities only with personal protective equipment and in the absence of animals.

### Limitations and future perspectives

Although the data obtained indicate the potential for using the complex preparation, several limitations should be acknowledged. Firstly, a limited number of bacterial strains were used in the microbiological experiments, which may underestimate the antimicrobial potential of DDAC-FAEO8. In future studies, we plan to expand the range of microorganisms tested. Secondly, because the toxicological studies aimed to assess the feasibility of using a complex preparation for disinfection of veterinary surveillance facilities, the test battery was limited to acute and subacute toxicity assessments and skin tests. However, assessment of chronic toxicity and genotoxicity is necessary and will be included in animal protocols. Finally, due to financial and time constraints, biodegradation experiments on the complex preparation and the assessment of its ecotoxicity were not conducted, thereby preventing the identification of potential environmental risks associated with the use of DDAC-FAEO8. Currently, data on these criteria are already known for DDAC. For example, DDAC is biodegradable under aerobic aquatic conditions (>70% in 28 days) and is stable for a considerable time (about 1 year) in soil [118]. Ecotoxicity data for DDAC compounds are available for 16 freshwater species of various trophic groups, including algae, fish, and invertebrates [18, 37]. Nevertheless, similar studies for the complex preparation are highly necessary and will be included in our future tasks. Despite the above limitations, the study provides a robust basis for further detailed investigations.

## CONCLUSION

The present study demonstrated that the combination of DDAC with FAEO8 significantly enhanced antimicrobial efficacy against *S. aureus*, *E. coli*, and *S*. Typhimurium compared to DDAC alone. The DDAC–FAEO8 formulation reduced MIC values and achieved >99% inhibition of microbial growth at ≥2× MIC, with prolonged bacteriostatic effects. Mechanistic investigations revealed that the enhanced activity was associated with increased membrane disruption, as evidenced by SEM observations, accelerated K^+^ leakage, and pronounced membrane depolarization. Although FAEO8 alone did not exhibit bactericidal activity, it contributed to increased membrane permeability, thereby facilitating DDAC action. Toxicological assessment indicated that the formulation exhibited low toxicity, with no mortality observed at doses up to 2000 mg/kg, minimal physiological alterations during subacute exposure, and only mild, reversible dermal irritation at working concentrations.

From a practical perspective, the improved antimicrobial performance combined with low toxicity suggests that the DDAC–FAEO8 formulation may serve as an effective disinfectant for veterinary sanitation, particularly in livestock facilities where both cleaning and disinfection are required. The ability to achieve high antimicrobial efficacy at lower effective concentrations may also reduce chemical load and environmental impact, addressing concerns associated with QAC accumulation and resistance development.

A major strength of this study is the integrated evaluation of antimicrobial activity and toxicological safety within a single experimental framework, supported by comprehensive mechanistic analyses. The use of multiple complementary methods, including MIC determination, IZD evaluation, SEM, K^+^ leakage, and membrane potential assays, provided strong evidence for the mode of action of the combined formulation.

However, certain limitations should be acknowledged. The study was conducted under controlled laboratory conditions, which may not fully replicate complex field environments encountered in livestock systems. In addition, the investigation was limited to selected bacterial strains, and the long-term ecological impact and potential for resistance development were not assessed.

Future studies should evaluate the performance of DDAC–FAEO8 under field conditions, including its efficacy in the presence of organic matter and diverse microbial communities. Further investigation into long-term safety, environmental fate, and resistance dynamics is also warranted. In addition, optimizing formulation ratios and assessing its efficacy against a broader spectrum of pathogens, including biofilm-forming microorganisms, would enhance its applicability.

In conclusion, the DDAC–FAEO8 combination represents a promising disinfectant formulation with enhanced antimicrobial efficacy and a favorable safety profile. Its dual functionality in cleaning and disinfection, coupled with low toxicity, supports its potential application in veterinary sanitation systems, although further validation under practical conditions is required.

## DATA AVAILABILITY

The supplementary data can be made available from the corresponding author upon request.

## AUTHORS’ CONTRIBUTIONS

OG, DG, AL, and NP: Conceptualized, designed, analyzed data, and revised the manuscript. GS, ES, NK, VT, VP, PT, and IV: Data collection, data analysis, interpretation, drafted the manuscript. NP and AL: Supervised and revised the manuscript. All authors have read and approved the final version of the manuscript.
